# Extracellular vesicles isolated from milk can improve gut barrier dysfunction induced by malnutrition

**DOI:** 10.1038/s41598-021-86920-w

**Published:** 2021-04-07

**Authors:** Mohamed Karim Maghraby, Bo Li, Lijun Chi, Catriona Ling, Abderrahim Benmoussa, Patrick Provost, Andrea C. Postmus, Abdirahman Abdi, Agostino Pierro, Celine Bourdon, Robert H. J. Bandsma

**Affiliations:** 1 Department of Nutritional Sciences, School of Graduate Studies, University of Toronto, Toronto, ON Canada; 2 Translational Medicine Program, Hospital for Sick Children, Toronto, ON Canada; 3 Centre de Recherche du CHU Sainte-Justine, Montreal, QC Canada; 4 Department of Microbiology-Infectious Disease and Immunity, Faculty of Medicine, CHUQ Research Center/CHUL, Université Laval, Quebec, QC Canada; 5 Faculty of Medical Sciences, University of Groningen, Groningen, The Netherlands; 6 KEMRI-Wellcome Trust Research Programme, Kilifi, Kenya

**Keywords:** Gastrointestinal models, Mouse, Intestinal stem cells, Malnutrition

## Abstract

Malnutrition impacts approximately 50 million children worldwide and is linked to 45% of global mortality in children below the age of five. Severe acute malnutrition (SAM) is associated with intestinal barrier breakdown and epithelial atrophy. Extracellular vesicles including exosomes (EVs; 30–150 nm) can travel to distant target cells through biofluids including milk. Since milk-derived EVs are known to induce intestinal stem cell proliferation, this study aimed to examine their potential efficacy in improving malnutrition-induced atrophy of intestinal mucosa and barrier dysfunction. Mice were fed either a control (18%) or a low protein (1%) diet for 14 days to induce malnutrition. From day 10 to 14, they received either bovine milk EVs or control gavage and were sacrificed on day 15, 4 h after a Fluorescein Isothiocyanate (FITC) dose. Tissue and blood were collected for histological and epithelial barrier function analyses. Mice fed low protein diet developed intestinal villus atrophy and barrier dysfunction. Despite continued low protein diet feeding, milk EV treatment improved intestinal permeability, intestinal architecture and cellular proliferation. Our results suggest that EVs enriched from milk should be further explored as a valuable adjuvant therapy to standard clinical management of malnourished children with high risk of morbidity and mortality.

## Introduction

Despite standardized inpatient treatment for acutely ill children with Severe Acute Malnutrition (SAM), mortality remains high and linked to about 500,000 deaths per year^1,2^. As per World Health Organisation guidelines, children are classified as having SAM if their weight-for-height is less than minus 3 z-score, and/or have a middle upper arm circumference less than 115mm^3^. Children with SAM, with or without additional complications, are also known to suffer from an enteropathy characterized by villus atrophy associated with nutrient malabsorption, intestinal inflammation, and increased intestinal permeability^4–6^. Infections are common in children with complicated SAM and are related to immune impairments and the lack of ability to maintain an effective intestinal barrier that could lead to bacterial translocation across the intestinal lumen and subsequent sepsis^7–9^. To this date, there are no effective treatment modalities targeting the repair of the intestinal epithelial barrier function in malnourished children.

Exosomes are small extracellular vesicles (EVs; 30-150 nm)^10^ that are stored within multivesicular bodies of cells, and are released upon fusion with the plasma membrane^11^. They are secreted by most, if not all cell types, and function as a novel mode of intercellular communication^11,12^. To reach target cells, released EVs can be transported by a variety of body fluids, including breast milk, and their lipid bilayer structure protects their internal molecular cargo which contains specific microRNA and a specialized functional proteome^11,13^. Once taken up by target cells, these exosomal factors are released and can elicit a variety of cellular processes (e.g. proliferation, cellular movement, apoptosis, or changes in molecular transport)^14^. Also, *in-vitro* models using intestinal epithelial cells and *in-vivo* mouse models have demonstrated that EV administration can improve epithelial cell viability and stimulate intestinal stem cell activity and proliferation^15,16^.

Some evidence suggests that milk-derived EVs can stimulate repair in intestinal injury models. Intestinal epithelial cells subjected to oxidative stress to simulate necrotizing enterocolitis (NEC) show significant improvements in viability when treated with breast milk-derived EVs, a demonstration of their protective effects and potential therapeutic applications^17,18^. In premature rat pups exposed to NEC inducing conditions, the administration of EVs significantly reduced disease severity^19–22^.

Our understanding of how milk-derived EVs can induce intestinal tissue repair remains very limited, especially in the context of malnutrition. We hypothesized that, in a mouse model of malnutrition, milk-derived EVs can, despite continued protein restriction, induce intestinal stem cell proliferation with restoration of intestinal architecture and improvement of intestinal epithelial barrier function.

## Results

### Extracellular vesicle enrichment and characterization

**Figure 1:**
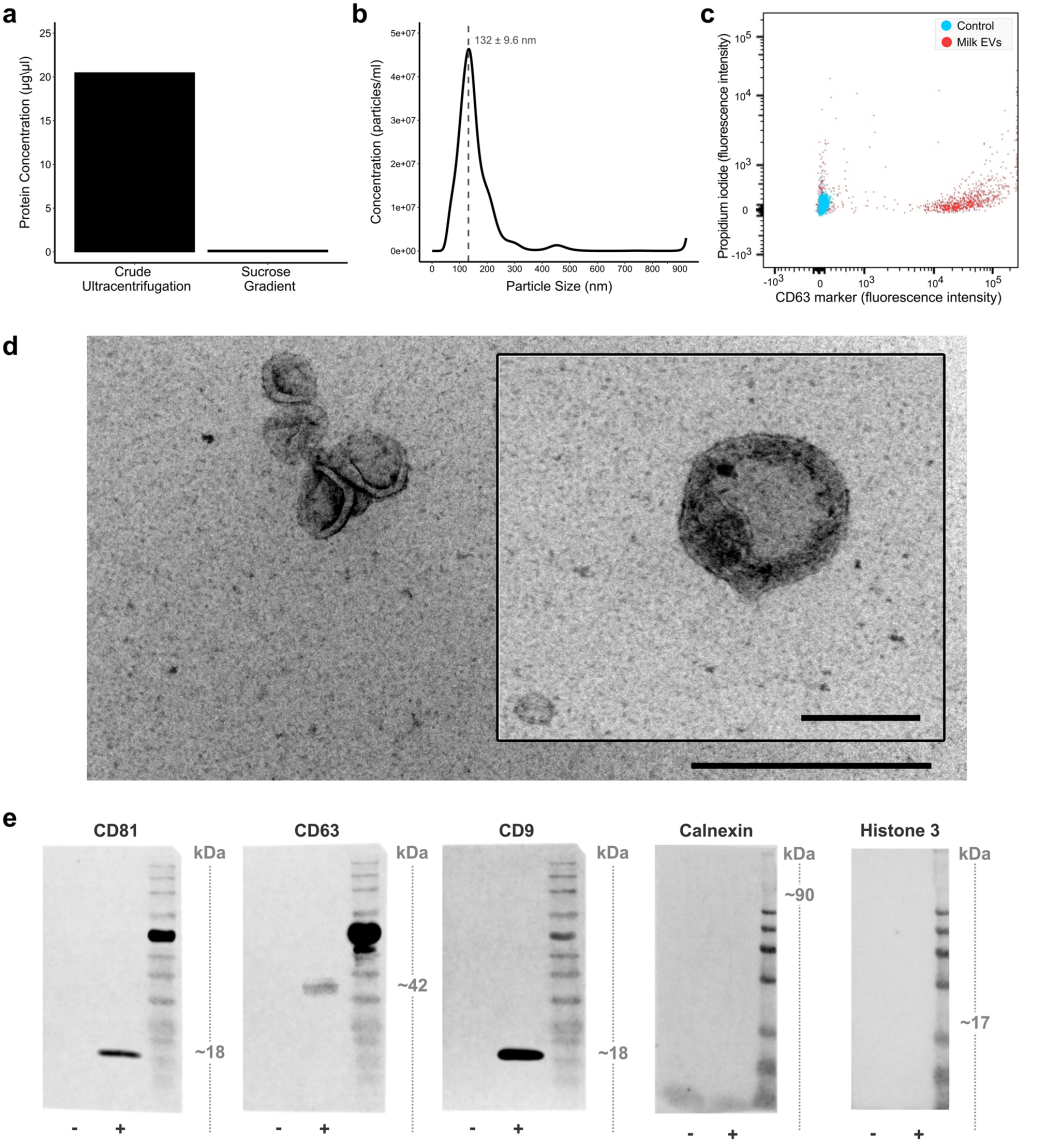
Enrichment and characterization of milk EVs. (**a**) Protein quantification of milk EVs enriched using (1) crude ultracentrifugation (20.48 µg/µl) and (2) ultracentrifugation with sucrose gradient purification (0.21 µg/µl). After ultracentrifugation with sucrose gradient, enriched milk EVs were pooled (n = 3) and characterised by: (**b**) Nanoparticle tracking analysis to demonstrate distribution of particle size and their concentration; (**c**) Flow cytometry to detect the milk EV-associated surface marker CD63 where the X-axis indicates fluorescence intensity linked to CD63, while the Y-axis plots fluorescence of propidium iodide relating to the presence of non-viable cells which are not expected after EV isolation procedures ; (**d**) Transmission electron microscopy to assess morphology, showing milk EVs at 62,000 × magnification (scale bar 500 nm) and a single milk EV at 100,000 × magnification (scale bar 200 nm);  (**e**) Full length immunoblots showing positive EV-membrane surface markers CD81, CD63 and CD9, and negative EV-markers calnexin and histone 3. Lanes indicated with negative symbol (−) are negative controls and positive symbol (+) indicate EV protein loading. Figures in panel a and b were generated with GraphPad Prism version 6.0.0 for Windows (www.graphpad.com); image for panel c was exported from FlowJo (www.flowjo.com).

**Figure 2:**
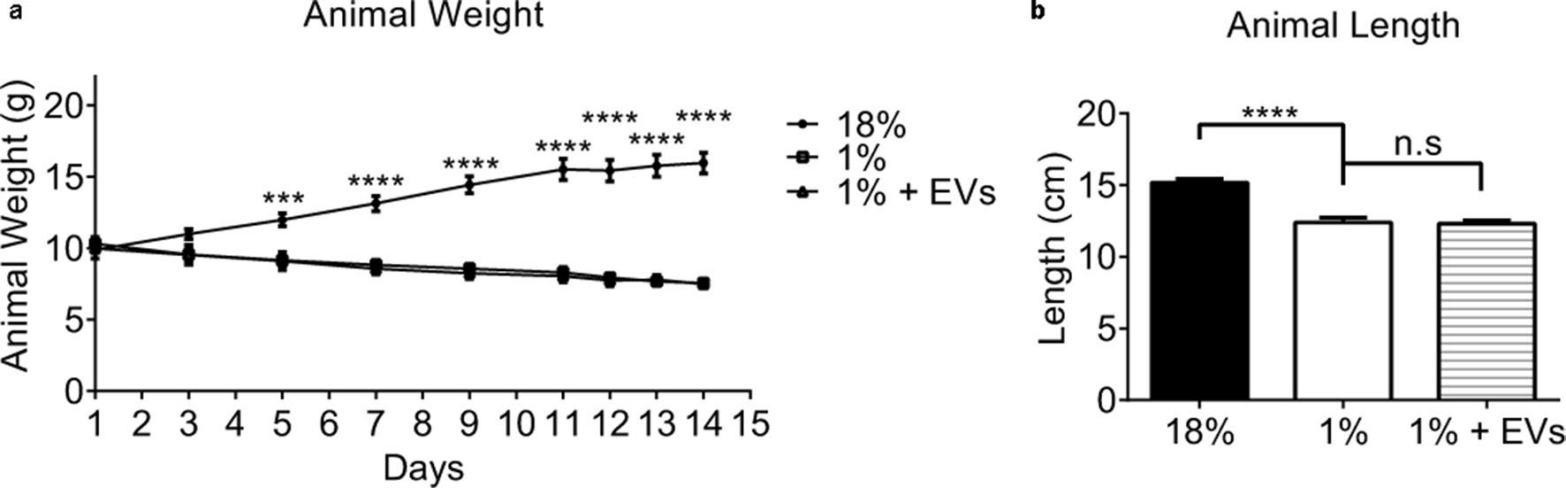
Impact of malnutrition and milk EV treatment on body measures of mice. (**a**) Body weight of sham-treated mice fed 1% protein diet was significantly lower at day 14 when compared to controls i.e. sham-treated mice fed 18% protein diet (n = 7, *P* < 0.0001). The body weights of milk EV-treated mice fed 1% protein diet did not improve compared to sham-treated mice fed 1% protein diet (n = 7, *P* = 0.72). (**b**) The body length at day 14 of sham-treated mice fed 1% protein diet was significantly shorter than controls i.e. sham-treated mice fed 18% protein diet (n = 7, *P* < 0.0001); and milk EVs treatment did not impact body length (n = 7). Data are presented as mean ± standard error of mean (SEM). Significance: ***P  < 0.001, ****P  < 0.0001, n.s = not significant. Graphs and statistical tests were done using GraphPad Prism version 6.0.0 for Windows (www.graphpad.com).

**Figure 3:**
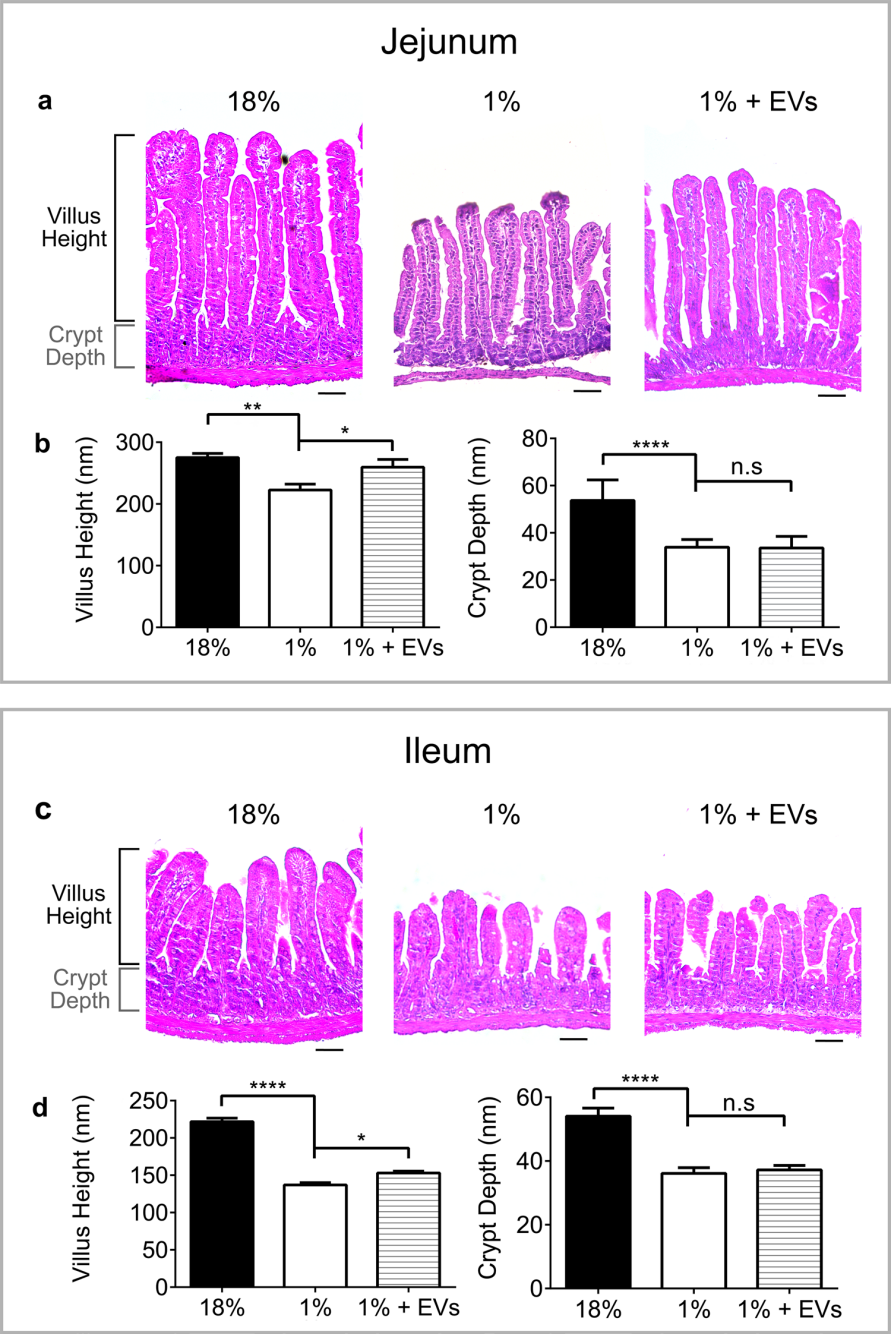
Milk EVs restore villus architecture in malnourished mice. Analysis of intestinal morphology of: controls, i.e., sham-treated mice fed 18% protein diet; sham-treated mice fed 1% protein diet; and milk EV-treated mice fed 1% protein diet: (**a**) H&E stained sections of jejunum and associated measurements of (**b**) Villus height and crypt depth, n = 7/group. (**c**) H&E stained sections of ileum and associated measurements of (**d**) Villus height and crypt depth, n = 7/group. Each column represents mean ± standard error of mean (SEM). (****P < 0.0001, **P  < 0.01, *P < 0.05, n.s = not significant), scale bar 50 nm. Graphs and statistical tests were done using GraphPad Prism version 6.0.0 for Windows (www.graphpad.com).

EVs derived from bovine milk (milk EVs) were first enriched by ultra-centrifugation and then further purified by sucrose gradient ultracentrifugation. The final protein concentration of enriched milk EVs was 0.21 µg/μl and calculated to have an insignificant nutritional contribution of 0.2% of protein to the overall daily intake of malnourished mice (Fig. 1a). Milk EV-enrichment was confirmed by: (1) nanoparticle tracking analysis which measured a peak particle size of ~ 132 ± 9.6 nm in diameter (Fig. 1b); (2) flow cytometry to detect the EV-associated surface marker CD63 (Fig. 1c); (3) transmission electron microscopy to visualize both the size and discoid morphology of milk EVs (Fig. 1d and Supplementary fig. S1); and (4) western blot to detect positive EV-membrane surface markers CD63, CD81, and CD9 and negative markers Calnexin and Histone 3 (Fig. 1e).

### Body changes in milk EV- and sham-treated malnourished mice

Malnutrition was induced in C57BL/6J mice by 1% protein diet feeding post-weaning (Fig. 2). Mice fed a 1% protein diet were significantly wasted and stunted at day 14 compared to sham-gavaged control mice kept on normal diet (controls) [Weight at day 14: 1% group, 7.6 ± 0.2 g vs 18% group, 16.0 ± 0.7 g, *P* < 0.0001 (Fig. 2a); Length at day 14: 1% group, 12.3 ± 0.3 cm vs 18% group, 15.4 ± 0.3 cm, *P* < 0.0001 (Fig. 2b)]. At day 14, milk EV-treated mice fed 1% protein diet weighed 7.5 ± 0.3 g (Fig. 2a) and measured 12.3 ± 0.2 cm in length (Fig. 2b). The 4 days of milk EV-treatment did not improve the wasted or stunted body condition of mice kept on 1% protein diet.

### Milk EVs restore intestinal epithelial architecture in 1% protein-fed mice

Morphometric analysis of histological sections of the jejunum and ileum was performed (Fig. 3). 1% protein diet feeding led to jejunal and ileal villus atrophy, where villus length was 223 ± 9.8 nm in 1% protein diet fed mice compared to 275 ± 7.4 nm in controls (*P* < 0.01) (Fig. 3a,b) whereas ileal villus length was 137 ± 3.1 nm in 1% protein diet fed mice compared to 222 ± 4.9 nm in controls (*P* < 0.0001) (Fig. 3c,d). Milk EV-treatment significantly improved villus height in both the jejunum (260 ± 12 nm) and ileum (153 ± 2.6 nm) compared to sham-treated mice fed 1% protein diet (*P* < 0.05) but, while the jejunal villi reached 94% of the height of controls, the ileal villi only attained 69%. Milk EV-treatment did not improve jejunal or ileal crypt depths (Fig. 3b,d). Despite continued 1% protein diet feeding, mice treated with milk EVs showed an improved intestinal architecture, and this was more specifically observed in the jejunum.

### Milk EVs improve intestinal epithelial permeability and barrier function

**Figure 4:**
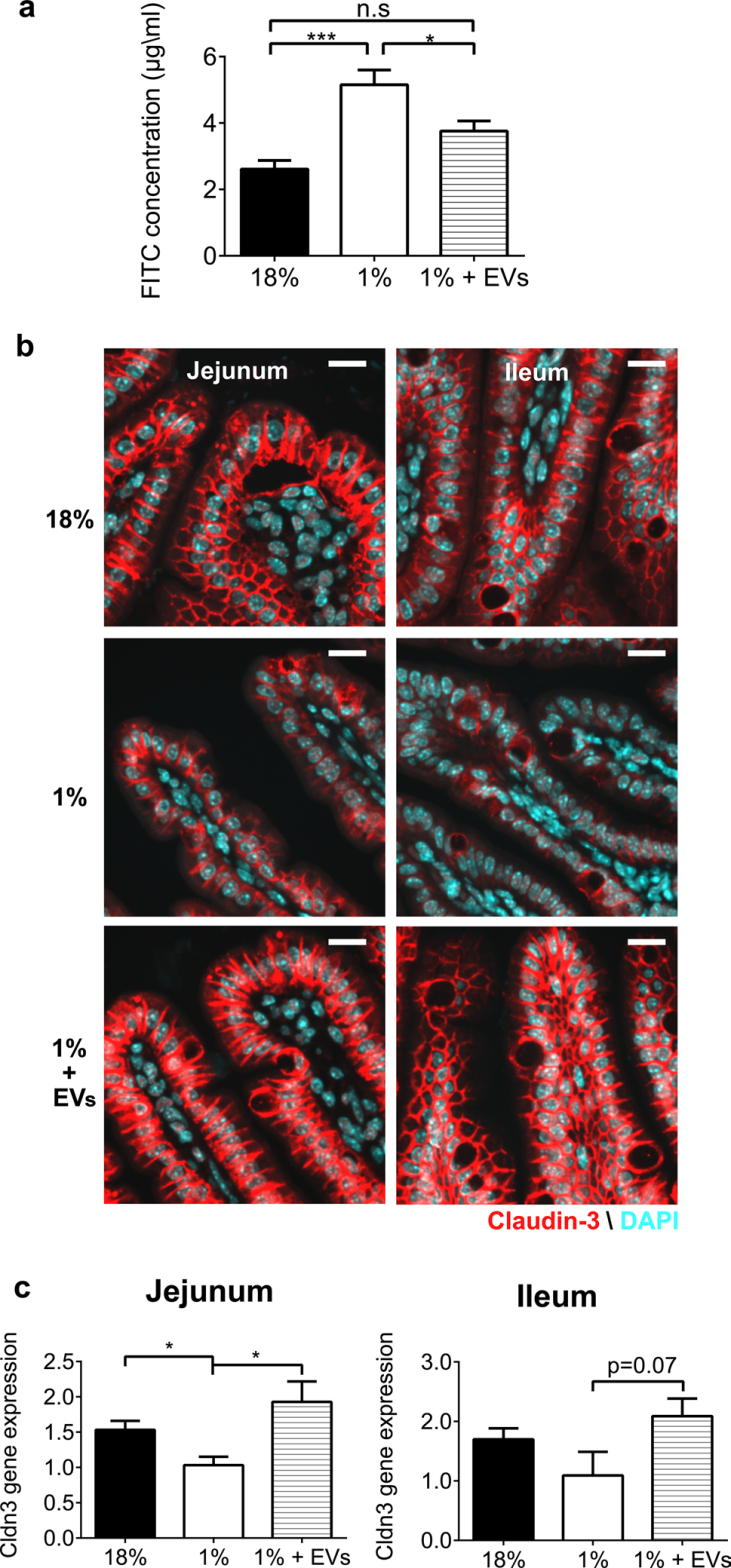
Milk EVs improve intestinal permeability and barrier protein claudin-3. Indicators of barrier function in controls, i.e. sham treated mice fed 18% diet; sham-treated mice fed 1% protein diet; and milk EV-treated mice fed 1% protein diet as represented by: (**a**) Fluorescein isothiocyanate dextran (FITC) assay of intestinal permeability (n = 7/group). Each column represents mean ± standard error of mean (SEM); (**b**) Immunofluorescent staining for intestinal epithelial barrier protein claudin-3 (red) in the jejunum and ileum with DAPI counterstaining of nuclei in blue, n = 3/group; 40 × magnification (Scale bar, 15 µm). (**c**) Claudin-3 (*Cldn3*) mRNA expression relative to ribosomal protein L13A *(Rpl13a)* in the jejunum and in the ileum (n = 6/group). Each column represents mean ± standard error of mean (SEM). *** P  < 0.001, * *P* < 0.05, n.s = not significant. Graphs and statistical tests were done using GraphPad Prism version 6.0.0 for Windows (www.graphpad.com).

Evaluation of intestinal epithelial permeability using Fluorescein Isothiocyanate (FITC) demonstrated that feeding a 1% protein diet led to an increase in serum FITC levels compared to control mice (5.15 ± 0.45 µg/ml vs 2.61 ± 0.26 µg/ml, *P* < 0.001) (Fig. 4a). This indicates that 1% protein diet feeding to young mice leads to intestinal epithelial barrier dysfunction. Notably, milk EV-treatment reduced FITC concentration in serum (3.76 ± 0.30 µg/ml) compared to sham-treated mice fed 1% protein diet (Fig. 4a; *P* < 0.05); where the concentration observed with milk EV-treatment did not differ from that of controls.

Immunofluorescent staining of claudin-3, a key protein in tight junction complexes and an indicator of barrier function^23^, showed that the 1% protein diet lead to a decrease in claudin-3 in the epithelial layer of the jejunum and ileum compared to control mice; whereas claudin-3 staining was higher in both the jejunum and ileum of milk EV-treated mice fed 1% protein diet compared to the sham-treated mice fed 1% protein diet (Fig. 4b). Concordantly, claudin-3 gene expression was also significantly reduced in the jejunum of 1% protein-fed mice compared to controls (*P* < 0.05); and milk EV-treatment restored its expression in the jejunum compared to sham-treated mice fed 1% protein diet (*P* < 0.05, Fig. 4c) while the ileum showed a similar trend (*P* = 0.07, Fig. 4c). We also probed for additional markers of tight junctions such as occludin and tight junction protein 1, also known as zonulin-1 (Zo-1) (Supplementary Fig. S2). When probing for occludin within the tissue, 1% protein diet feeding led to a reduction in protein staining which was not improved by milk EV-treatment (Supplementary Fig. S2a). However, occludin gene expression was down regulated in sham-treated mice fed 1% protein diet and expression was improved with milk EV-treatment (Supplementary Fig. S2b). No differences in *Zo-1* gene expression were found between groups (Supplementary Fig. S2c). Thus, milk EV-treatment significantly improved measures of intestinal permeability which is, at least in part, linked to the modulation of certain components of tight junctions and repair of intestinal architecture.

### Milk EVs improve proliferation of intestinal epithelial cells during malnutrition

**Figure 5:**
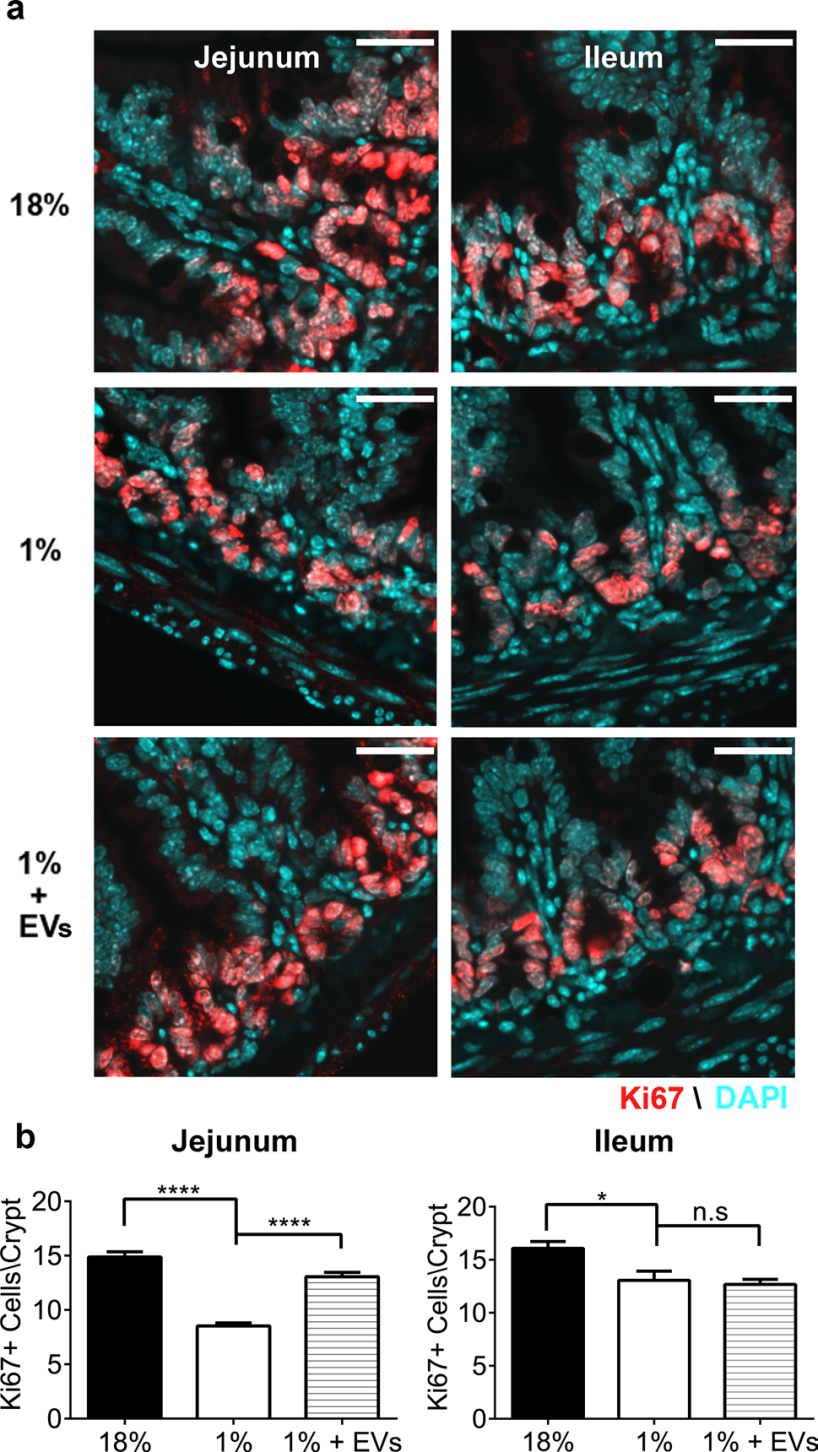
Milk EVs increase the number of proliferating Ki67 + cells in intestinal crypts in malnutrition. Number of proliferating cells in: controls i.e., sham-treated mice fed 18% protein diet; sham-treated mice fed 1% protein diet; and milk EV-treated mice fed 1% protein diet as represented by: (**a**) Immunofluorescent staining for proliferation marker Ki67 (red) in the jejunum and in the ileum with DAPI counterstaining of nuclei in blue; 40 × magnification (Scale bar, 25 µm) (**b**) Average count of Ki67 + cells per crypt in the jejunum and in the ileum. Each column represents mean ± standard error of mean (SEM) (****P < 0.0001, * P  < 0.05, n.s = not significant, n = 5/group). Graphs and statistical tests were done using GraphPad Prism version 6.0.0 for Windows (www.graphpad.com).

Investigation of cellular proliferation using marker of proliferation Ki67 (Fig. 5) demonstrated that feeding mice a 1% protein diet lead to a significant reduction in proliferating cells in intestinal crypts compared to control mice in both the jejunum and ileum (Fig. 5a) and that milk EV-treatment restored this expression in the jejunum. The jejunum of 1% protein diet mice showed 8.5 ± 0.3 positive cells/crypt compared to 14.9 ± 0.5 positive cells/crypt in 18% protein diet mice (*P* < 0.0001) (Fig. 5b), while the ileum showed that mice fed 1% protein diet had 13.0 ± 0.9 positive cells/crypt compared to 16.1 ± 0.7 positive cells/crypt in 18% protein diet mice (*P* < 0.05). Milk EV-treatment significantly increased cellular proliferation in mice fed 1% protein diet , evident by an increase in the number of Ki67 positive cells per crypt compared to sham-treated mice fed 1% protein diet in the jejunum (13.1 ± 0.3 positive cells/crypt, *P* < 0.0001, Fig. 5b). No difference was seen in the ileum. Thus, the 4 days of milk EV-treatment restored cellular proliferation in the intestinal crypts of the jejunum.

### Milk EVs induce *Lgr5* + stem cell proliferation and activation of Wnt pathway

**Figure 6:**
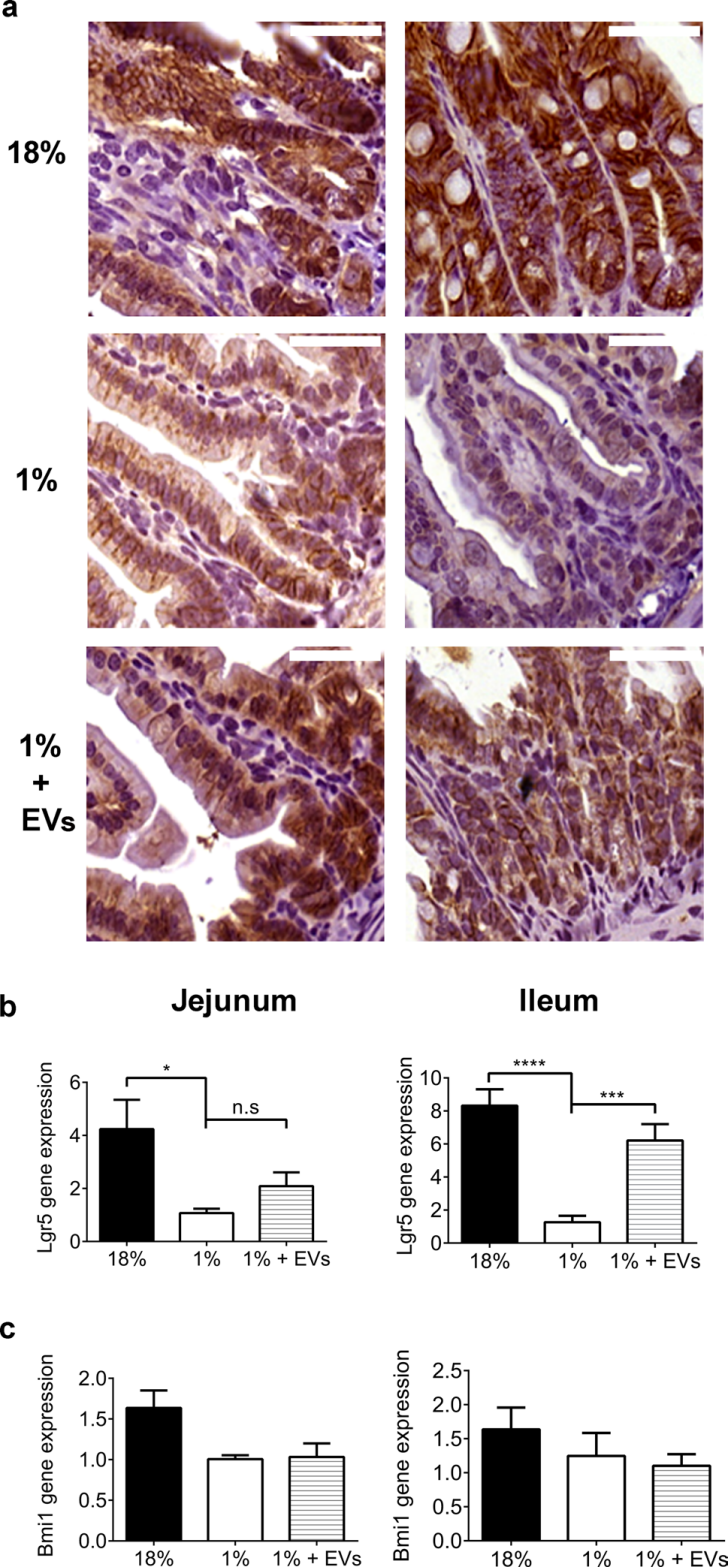
Milk EVs activate Wnt pathway, as seen by increased β-catenin staining, and intestinal epithelial stem cell proliferation, as seen by increased *Lgr5* + cells. Stem cell activation in: controls, i.e. sham-treated mice fed 18% protein diet; sham-treated mice fed 1% protein diet; and milk EV-treated mice fed 1% protein diet, represented by: Immunohistochemistry of (**a**) Jejunum and ileum staining for β-catenin, 63 × magnification, n = 3/group. Scale bar, 40 µm; (**b**) qPCR analysis of *Lgr5* expression relative to Ribosomal protein L13A (*Rpl13a*) in Jejunum and Ileum (****P < 0.0001, ***P < 0.001, n = 6/group). (**c**) qPCR analysis of *Bmi1* in jejunum and ileum relative to *Rpl13a* (n = 6/group). Each column represents mean ± standard error of mean (SEM). Graphs and statistical tests were done using GraphPad Prism version 6.0.0 for Windows (www.graphpad.com).

To explore the mechanism through which milk EVs induce an increase in cellular proliferation in mice fed 1% protein diet, immunohistochemistry (IHC) was performed for β-catenin, a downstream product of the Wnt pathway. Mice fed a 1% protein diet showed lower nuclear staining of β-catenin compared to controls (Fig. 6a), but milk EV-treatment increased nuclear staining of β-catenin compared to sham-treated mice fed 1% protein diet .

The effect of milk EVs on stem cell activity was assessed by analysing gene expression of two stem cell markers: (1) leucine rich repeat containing G protein-coupled receptor 5 (*Lgr5*) that marks actively dividing cells (Fig. 6b), and (2) BMI1 proto-oncogene, polycomb ring finger (*Bmi1*) that marks quiescent stem cells (Fig. 6c). Mice fed 1% protein diet demonstrated lower levels of *Lgr5* and *Bmi1* gene expression compared to control mice (*Lgr5* + : jejunum *P* < 0.05, ileum *P* < 0.0001; *Bmi1* + : jejunum *P* < 0.05, ileum n.s.). Consistent with our IHC findings, LGR5 protein expression significantly increased with milk EV-treatment compared to sham-treated mice fed 1% protein diet (ileum, *P* < 0.001 Fig. 6b). BMI1 protein expression did not show any significant difference with milk EV-treatment compared to sham-treated mice fed 1% protein diet . This suggests that milk EVs may not impact the quiescent stem cells but act by stimulating the active proliferation of *Lgr5* + stem cells and by maintaining Wnt pathway signaling.

## Discussion

**Figure 7:**
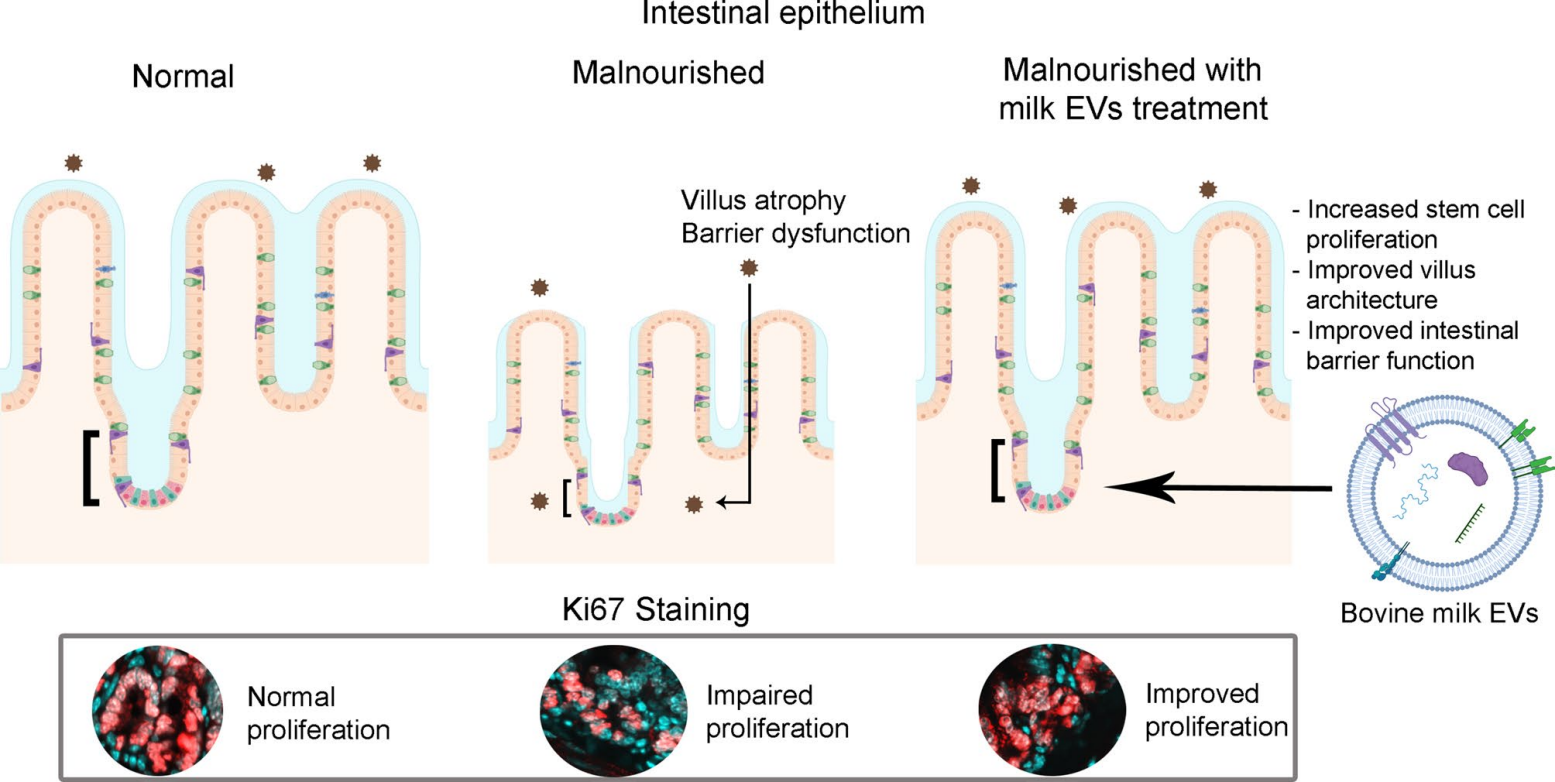
Conceptual model. Framework representing a mode of action of milk EVs in the restoration of barrier dysfunction and the repair of structural injury seen in the small intestinal villi of malnourished animals. Milk EV-treatment induced repair, at least in part, through increased stem cell proliferation in intestinal crypts. This figure was in part created with BioRender and was licenced for use in publication (created with BioRender.com).

Our study aimed to assess the effect of milk EVs on intestinal epithelial atrophy and barrier dysfunction in a mouse model of severe malnutrition. To our knowledge, this study is the first to show that the administration of milk EVs leads to a significant improvement in the architecture of the small intestine and the restoration of intestinal epithelial barrier dysfunction induced by 1% protein feeding (Fig. 7). Our results suggest that the benefits of milk EVs are linked to the modulation of certain components of tight junctions and the stimulation of cellular proliferation in the intestinal crypts and that improvements are observed despite mice being kept on a protein deficient diet throughout milk EV-treatment.

In children, malnutrition is known to increase intestinal permeability and impact the architecture of their small intestine causing mucosal atrophy and blunting of intestinal villi^4,6,24–26^ and this has been associated with reduced nutrient absorption^27,28^. Several malnutrition models in animals have been developed, and most used a variation of low protein diets^25,29–33^. For example, Smith et al. made use of a diet designed to mimic a typical protein deficient Malawian diet^34^. We used a low 1% protein diet to induce malnutrition in C57BL/6J mice post weaning and, as seen in humans, malnourished mice were wasted, stunted and showed increased intestinal permeability with signs of damage to their intestinal epithelium, namely, villus atrophy and blunting, which supports our dietary model of malnutrition.

In our study, intestinal cell proliferation was decreased in mice fed 1% protein diet as assessed by Ki67 staining, which could, at least in part, explain the blunting of the intestinal villi in malnutrition. The administration of milk EVs increased intestinal cell proliferation, despite continued 1% protein feeding, and this was associated with a restoration of intestinal architecture including increased villus length. Previous publications demonstrated evidence that EVs including exosomes, can promote the activity of stem cells when delivered to the intestinal epithelium^15,16,21^, which is consistent with our findings showing increased gene expression *of Lgr5* and an associated increase in *Ki67* + cells suggesting increased proliferation in intestinal crypts. It has been reported that exosomes stimulate *Lgr5* in intestinal crypts via induction of Wnt signaling pathway^16,35^, and, concordantly, we also observed changes in the Wnt signaling pathway. The mechanisms through which milk EVs interact and change the cellular behaviour of intestinal target cells remain poorly understood. Milk EVs proteins include tetraspanins, such as CD63^36^, which can interact with integrins and promote uptake by target cells^37^. Integrins interact with intercellular adhesion molecules and extracellular matrix (ECM) proteins, which may explain exosome specificity towards certain tissues and organs^38–41^. EVs may induce responses in recipient cells through surface membrane interaction^11,42^ or become internalized by clathrin-mediated or clathrin-independent mechanisms (phagocytosis, pinocytosis)^43–45^ and release their functional contents including microRNA in the cytoplasm of target cell^46–49^.

Our study is also the first to demonstrate the ability of milk EVs to improve intestinal epithelial permeability in a mouse model of malnutrition. As evaluated using serum measures of FITC, an established method for investigating the integrity of intestinal barrier function^50–53^, mice fed 1% protein diet showed marked intestinal barrier dysfunction, consistent with reports from previous studies in murine models^53^. Importantly, milk EV-treatment significantly improved this measure of intestinal permeability. The beneficial effect could be explained by the combined improvement in intestinal cellular architecture linked to increased stem cell proliferation and repair, together with structural changes in certain components of tight junctions as suggested by increased expression of Claudin-3 protein. Tight junctions control permeability across the epithelium of the intestine^54,55^ and, Claudin-3 is a major component that determines the morphology of tight junction strands^23^. While we explored other markers involved in the regulation of barrier permeability (i.e., *Zo-1* and occludin), we did not find evidence of improved protein levels with milk EV-treatment within the small intestine. Intestinal epithelial tight junction proteins are dynamically regulated in response to multiple factors, physiological and pathological. Their regulation mechanisms are not yet well understood^56^. This is also complicated by the non-uniform response of tight junctions to pathological conditions^53^. Thus, these results offer a possible regulation mechanism linked to improved barrier function with milk EV-treatment, but a deeper understanding would be gained from a more comprehensive investigation of this process.

Bovine milk was chosen as the raw material from which we extracted milk EVs for the following reasons. Firstly, milk is relatively accessible in most settings, including low- and middle-income countries. Secondly, extracting EVs from milk does not require the addition of any chemicals, preservatives or pharmaceutical components. Thirdly, emerging studies comparing the proteome of bovine and human breast milk derived EVs showed their significant overlap^57^. Furthermore, bovine and human milk microRNA profiles were found to be largely similar and conserved^49^. As discussed by Yang *et al*^57^, comparing the similarities between human and bovine milk EVs is of growing interest as it could aid in improving milk formulas given to infants. All these considerations are important logistic and heuristic advantages for their possible introduction into clinical trial settings.

In agreement with our study, the use of sucrose density gradient to enrich EVs from milk has been validated in a multitude of publications^58–60^ and proved to be a superior method compared to using commercial kits, which: (1) do not exclude protein or macromolecular contamination, (2) cannot discriminate between EVs based on size, and (3) have been up to date only tested on plasma and cell culture media^61–63^. In this study, we chose a 4 day treatment regimen which theoretically allows for one complete cycle of cellular turnover in the intestinal epithelium^64^, however, the local dynamics of cell turnover and repair likely differ in conditions of malnutrition. Thus, although we observed improvements in intestinal architecture and function, it is foreseeable that more extensive repair could be achieved with longer treatment duration.

Limitations of this study include that mice in our model were only subjected to dietary insult whereas children suffering from severe complicated malnutrition have concomitant infections, intestinal inflammation and often suffer from diarrhea, which adds to the burden of morbidity. Thus, we do not know whether milk EVs would significantly improve these comorbidities. While our analysis explored milk EV-mediated mechanisms that can induce intestinal repair, their specific functional components were not here identified. Also, we did not test whether nutrient absorption is impaired in our model and if milk EV treatment could restore any defects in absorption. Other doses and treatment regimens should be tested if longer term effects on weight and/or length are to be revealed. Our study focused here on the small intestine, but colonic effects of both 1% diet feeding and EV-treatment could also be evaluated in future studies. We measured intestinal epithelial barrier permeability using a common approach based on FITC-dextran but this methodology could be strengthened by varying the time of blood collection to assess specific segments of the intestine, using molecular probes of different diameters to target different leak pathways and by using complementary approaches such as FITC-Inulin^52,58,59^. We also did not evaluate gender differences in our study, however, malnutrition and malnutrition-related enteropathy does not clinically affect male or female children differently. In addition, when we first characterized the effects of the dietary insult in C57BL/6J mice, we found no gender differences in the phenotypic impact of the 1% diet on the small intestine (data not shown). This decision was also taken to reduce experimental animal usage and costs. Lastly, both milk EVs and malnutrition are known modulators of immune function; thus, the interactive effect on systemic inflammation and immune activation would also be key questions to answer.

In summary, we demonstrate that the administration of milk EVs in a mouse model of malnutrition leads to a significant improvement of intestinal dysfunction, and this should be explored further as a potential therapeutic intervention in the clinical setting to help improve the outcomes of children with malnutrition and intestinal dysfunction.

## Methods

### Animals

Animal work was performed in accordance with the Canadian Council on Animal Care guidelines using protocols approved by the Animal Care Committee of The Hospital of Sick Children, Toronto ON. (Animal Use Protocol Number 1000030900). Male C57BL/6J mice (Jackson Laboratories, Bar Harbor, Maine USA) were weaned at three weeks post-partum, weight-matched and randomized into either a control group (n = 7) fed a normal diet with 18% protein (Teklab Custom Diet TD.10098, Envigo, Madison, Wisconsin, USA), or a malnourished group (n = 7) fed an isocaloric low protein diet (1% protein Teklab Custom Diet TD.150358, Envigo), or a malnourished treatment group (n = 7) fed with the protein deficient diet and designated to receive treatment with milk EVs. Dietary intervention lasted 14 days, with body weights collected every other day, and the aforementioned mice groups received a daily oral gavage of milk EVs (dose: 4.83e + 006 ± 1.68e + 005 milk EVs per g of body weight) or sham treatment of phosphate buffered saline (PBS) from Days 10 to 14 with body weights collected every day for the last 4 days. Animal length measurements were done at time of sacrifice. As the average time required for the intestinal epithelium to regenerate is 3–5 days, a conservative treatment duration of 4 days was selected to allow for one cycle of epithelium regeneration to occur. We selected the dose of milk EV based on previous experience^60^. The dosing regimen also considered existing guidelines for oral gavage in experimental mice, which are based on animal weight^61^ and gavage was restricted to once a day to minimize stress that could differentially impact weaker malnourished animals.

### Tissue collection and processing

Mice were sacrificed on day 15 post weaning. Food and water were removed 6 h before necropsy. To assess intestinal permeability, 4-Kda FITC-labelled dextran (Sigma-Aldrich, Oakville, Ontario, Canada) prepared at a standard concentration of 100 mg/ml diluted in sterile PBS was administered to mice via oral gavage at a dose of 6 µl/gbw, 4 h before blood collection by cardiac puncture. Blood was collected in EDTA tubes (Becton, Dickenson and Company, Franklin lakes, New Jersey, USA) and placed on ice. Serum was obtained by spinning at 2500 g for 20 min using a lab bench centrifuge (Eppendorf, Hamburg, Germany). For FITC measurement, serum was then diluted with a 1:1 to 1:3 dilution factor using 1X PBS depending on the amount of serum available and pipetted in triplicates on a 96-well plate (Corning, Corning, New York, USA). FITC concentration was measured using a fluorescent plate reader (SpectraMax Gemini EM, Molecular Devices, San Jose, California, USA), calibrated at excitation of 485 nm and emission of 528 nm. Measurements were calculated against a standard curve of FITC dilutions done from the original preparation used to gavage the mice. Results were obtained and analyzed with in-built SoftMax Pro Software version 5 (Molecular Devices).

The intestine was harvested and oriented to approximate anatomical segments. The medial/jejunum and distal/ileum portions of the small intestine were collected and flushed with ice cold PBS. All tissues were collected on ice and intestinal segments were divided into two parts. The first part was fixed overnight in 10% Formalin (Thermo Fisher Scientific, Waltham, Massachusetts USA) and the second was frozen on dry ice and stored in -80 °C for RNA extraction.

### Isolation of milk EVs

Milk EVs were enriched from locally sourced fresh unpasteurized bovine milk using differential ultracentrifugation (adapted from protocol by Chiou N. & Ansel K.M)^62^. Milk aliquots were subjected to a series of centrifugations to remove fats, cells and cellular debris, filtered through grade 1 Whatman filter paper (Sigma-Aldrich), and finally passed through 0.2 µm syringe filter (FroggaBio, Toronto, Ontario, Canada).

Milk was transferred to polyvinyl-carbonate ultracentrifuge tubes (Beckman Coulter, Brea, California, USA), mounted on SW 32 Ti swivel rotor (Beckman Coulter), and ultracentrifuged at 100,000 g for 13 h at 4 °C (Optima Ultracentrifuge LE-80 K, Beckman Coulter) to pellet milk EVs. The pellet was reconstituted with PBS and mixed 1:1 with 90% sucrose diluted in sterile PBS and set at the bottom of an ultracentrifuge tube immersed in ice. Thereafter, a sucrose gradient was created by layering decreasing concentrations of sucrose solution (70, 64,58,52,46,40,34,28,16 and 10%). The sample was ultracentrifuged at 100,000 g for 16hrs at 4 °C. Thereafter, the layer enriched with milk EVs was collected, washed with PBS and centrifuged at 100,000 g for 1 hr at 4 °C. Samples of milk EVs were pooled and proteins quantified using Pierce BCA Protein Assay Kit according to manufacturer protocol (Thermo Fisher Scientific). Particle concentration was determined by Nanoparticle Tracking Analysis (NTA) (NanoSight LM10, Malvern Panalytical, Malvern, UK). Briefly, 1 ml samples of milk EVs were manually analysed using 3 acquisitions in 40 s intervals from 5 separate fields of view; this standard NTA routine used a 65 mWatt 405 nm violet laser, sCMOS camera (camera level 5, slider shutter 45 and gain of 15) and NanoSight NTA Software Build 3.1.46 (Malvern Panalytical).

Milk EV enrichment was confirmed by visualization using transmission electron microscopy (Philips CM10 Transmission Microscope, Field Electron and Ion Company/Thermo Fisher Scientific, Hillsboro, Oregon, USA) and by flowcytometry to detect milk EV surface marker CD63. Briefly, EVs were incubated with CD63 antibody at 4˚C overnight and then incubated with FITC secondary antibody for 2 h at room temperature, washed and analyzed by flow cytometry using a Gallios Flow Cytometer (Beckman Coulter) and compared with a sample not incubated with CD63 primary antibodies. Data was analyzed using FlowJo Software version 10^63^. Western blots were done using ExoA Antibody Kit according to the manufacturer’s protocol (System Biosciences, Palo Alto, California, USA). Immunoblots were cut prior to primary antibody hybridization. Immunopositive bands were detected using ECL Plus Kit (Invitrogen, Carlsbad, California, USA) according to manufacturer’s protocol. Protein band intensity was captured using Odyssey Fc (LI-COR Bioscience, Lincoln, Nebraska, USA) and the level of protein expression was quantified using Image Studio 5 (LI-COR). Images provided are the largest view saved from image acquisition.

### Histological processing and intestinal morphometry

Formalin fixed segments of the jejunum and ileum (~ 1 cm in length) were dehydrated with ethanol, segmented, and embedded in paraffin wax to produce 5 µm thick cross-sections and stained with Hematoxylin and Eosin. Stained sections were examined under light microscope and images were captured using a mounted camera (Leica Microsystems, Wetzlar, Germany). Villus height and crypt depth were measured using ImageJ Software (https://imagej.nih.gov/ij/index.html). Five intact, well-oriented villi per slide were measured from the apex to the villus-crypt junction. Crypt depth measurement was performed in a similar manner – five, well-visualized, well-oriented crypts were measured from the crypt-villus junction to the base of the crypt. All morphometric analysis was repeated by a blinded researcher.

### Immunofluorescence and immunohistochemistry

Paraffin wax slides were stained for Ki67 to investigate cellular proliferation (anti-rabbit Ki67 antibody, Abcam, Cambridge, U.K.) and for claudin-3 for changes in barrier function (anti-rabbit claudin-3 antibody, Thermo Fisher Scientific). Slides were mounted with DAPI-containing medium (Prolong Gold Antifade with DAPI, Thermo Fisher Scientific) and images captured using a spinning disc confocal microscope (Nikon Canada, Mississauga, Ontario, Canada). For Ki67, the average of five intestinal crypts for each sample was counted to calculate the mean.

Nuclear β-catenin was visualized using IHC to investigate Wnt pathway activation. IHC was performed on paraffin wax embedded tissue sections using a two-step EnVision/HRP technique (Agilent, Santa Clara, California, USA) following manufacturer’s protocol.

### RNA isolation and real-time qPCR

Epithelial scrapings obtained from frozen segments of the jejunum and ileum were used for RNA extraction with Trizol reagent following manufacturer’s protocol (Direct-zol RNA Kits, Zymo Research, Irvine, California, USA). RNA concentration was determined using a NanoDrop 2000 spectrophotometer (Thermo Fisher Scientific). Then, 1 µg of RNA was added to a reverse transcription reaction using qScript cDNA Synthesis Kit (Quantabio, Beverly, Massachusetts, USA) according to manufacturer’s protocol.

Real-time PCR was performed in triplicates using 0.1 µl cDNA per reaction with SYBR Green PCR Master Mix (Thermo Fisher Scientific) and primers listed below (Table 1). The reaction was performed on a C1000 Touch Thermal Cycler equipped with CFX384 Real Time System (Bio-Rad Laboratories, Hercules, California, USA). Ribosomal protein L13A (*Rpl13a*) was used as an endogenous control for normalization. All primers listed below were verified in nucleotide BLAST database and purchased from Integrated DNA technologies (IDT, Coralville, Iowa, USA).

**Table 1:** Real-time PCR primer sequences.

Gene	Forward and Reverse primer sequence
*Bmi1*	F: CCAATGAAGACCGAGGAGAA R: TTTCCGATCCAATCTGCTCT
*Cldn3*	F: ACCAACTGCGTACAAGACGAC R: CGGGCACCAACGGGTTATAG
*Lgr5*	F: CGAGCCTTACAGAGCCTGATACC R: TTGCCGTCGTCTTTATTCCATTGG
*Rpl13a*	F: TCCCTCCACCCTATGACAAG R: GTCACTGCCTGGTACTTCC
*Zo-1*	F: GGAGATGTTTATGCGGACGG R: CCATTGCTGTGCTCTTAGCG
*Occludin*	F: CACTGCACCCTGAGAAGCAT R: CGAGCCTCCTTAGCTCGTAG

*Bmi1* B cell-specific Moloney murine leukemia virus integration site 1, *Lgr5* leucine-rich repeat-containing G-protein coupled receptor 5, *Rpl13a* Ribosomal protein L13A, *Zo-1* Zonulin-1.

### Statistical analysis

All results are expressed as mean ± standard error of mean (S.E.M). Statistical significance between groups was calculated using ordinary one-way analysis of variance (ANOVA) with Tukey’s post-hoc analysis. Weight differences were assessed with repeated measures ANOVA. Statistical analysis was performed using GraphPad Prism version 6.0.0 for Windows (GraphPad Software, San Diego, California, USA, www.graphpad.com) and statistical significance was considered whenever *P  < 0.05; **P < 0.01; ***P < 0.001; ****P < 0.0001. Image processing and collages were done using Volocity 6.5.1 Windows (https://quorumtechnologies.com/volocity/volocity-downloads/downloads), ImageJ (https://imagej.nih.gov/ij/download.html), Inkscape (https://inkscape.org) and Adobe Photophop (www.adobe.com). Conceptual model figure created using www.BioRender.com.

## Supplementary Information

Supplementary Information
