# Supplementary material for: Extracellular vesicles isolated from milk can improve gut barrier dysfunction induced by malnutrition
Source: Sci Rep. 2021 Apr 7;11:7635. doi: 10.1038/s41598-021-86920-w (PMC8026962; doi:10.1038/s41598-021-86920-w)
Supplement: Supplementary file 1 — Supplementary Information [file 41598_2021_86920_MOESM1_ESM.docx]

**Extracellular Vesicles Isolated From Milk Can Improve Gut Barrier Dysfunction Induced By Malnutrition**

**Mohamed Karim Maghraby^1^, Bo Li^2^, Lijun Chi^2^, Catriona Ling^1^, Abderrahim Benmoussa^3^, Patrick Provost^4^, Andrea Postmus^5^, Abdirahman Abdi^6^, Agostino Pierro^2^, Celine Bourdon^2^, Robert HJ Bandsma*^2^.**

1. Department of Nutritional Sciences, School of Graduate Studies, University of Toronto, Toronto, Ontario, Canada, 2. Translational Medicine Program, Hospital for Sick Children, Toronto, Ontario, Canada, 3. Centre de recherche du CHU Sainte-Justine, Montreal, Quebec, Canada, 4. CHUQ Research Center/CHUL, Department of Microbiology-Infectious Disease and Immunity, Faculty of Medicine, Université Laval, Quebec, Quebec, Canada, 5.Faculty of Medical Sciences, University of Groningen, Groningen, Netherlands, 6. KEMRI-Wellcome Trust Research Programme, Kilifi, Kenya.

Supplementary information

**Supplementary figure S1**

**
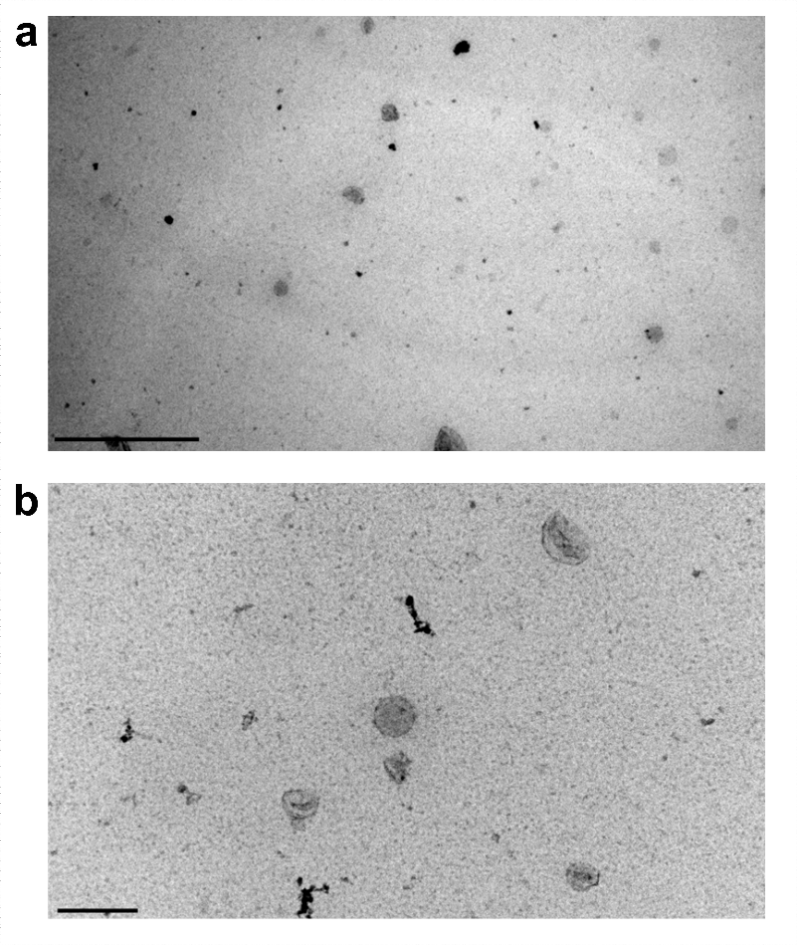
**

**Supplementary figure S1:** Milk EVs visualized with transmission electron microscopy at (**a**) 29,000x magnification (scale bar = 1 µm) and (**b**) 80,000x magnification (scale bar = 200 nm).

**Supplementary figure S2**


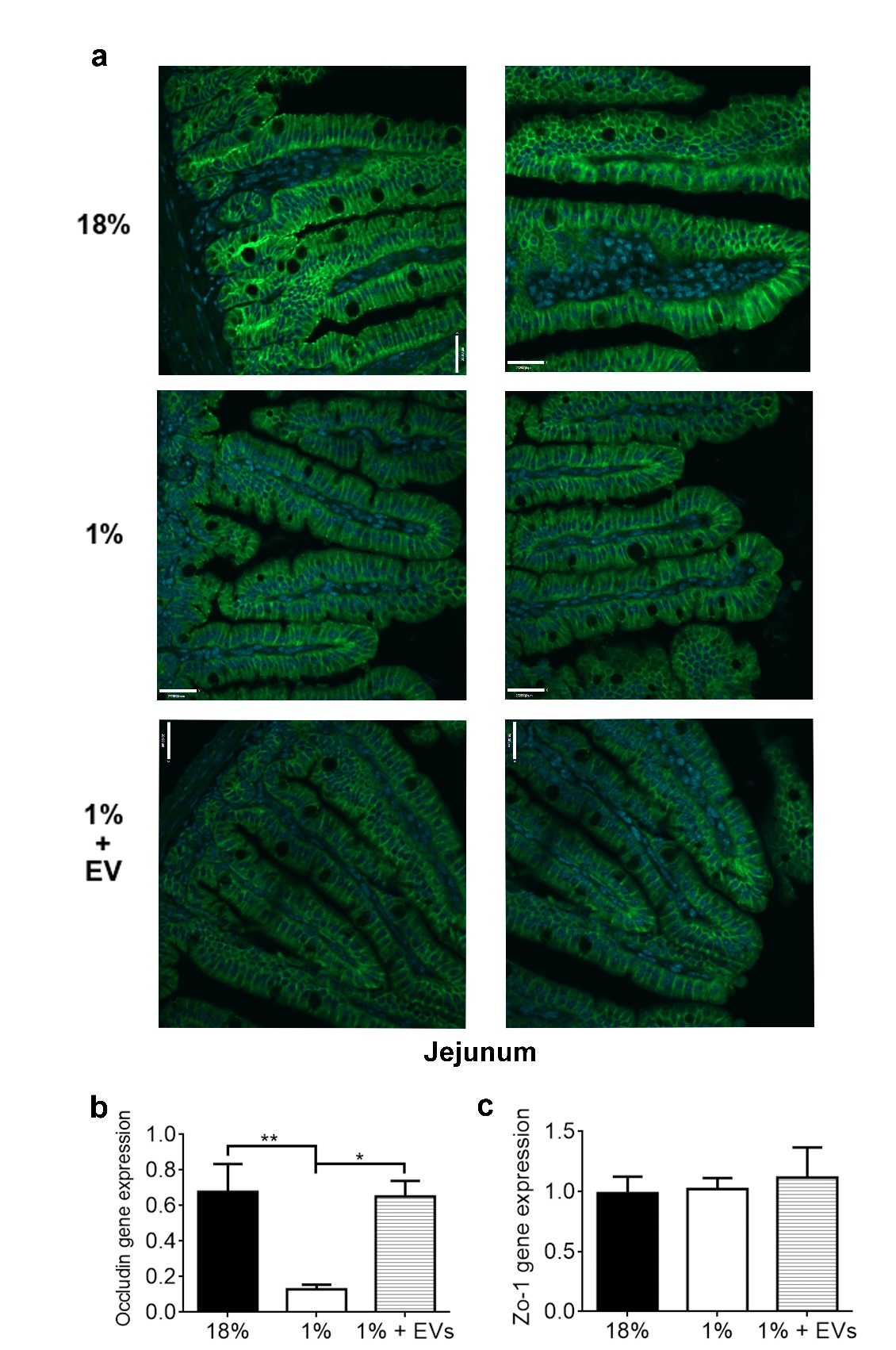


**Supplementary figure S2:** Effects of milk EVs on additional indicators of intestinal barrier function in: controls, i.e. sham-treated mice fed 18% protein diet; sham-treated mice fed 1% protein diet; and milk EV-treated mice fed 1% protein diet. (**a**) Immunofluorescent staining of occludin (green) in the jejunum with DAPI counterstaining of nuclei in blue, n=3/group; 20x magnification (Scale bar, 28µm). occludin and zonulin-1. Gene expression changes of (**b**) occludin and (**c**) *Zo-1* mRNA expression relative to *Rpl13a* in the jejunum (n=6/group). Each column represents mean ± standard error of mean (SEM). **p<0.01, * p<0.05.
